# Supplementary material for: Integrated Bioinformatics and Validation Reveal IFI27 and Its Related Molecules as Potential Identifying Genes in Liver Cirrhosis
Source: Biomolecules. 2023 Dec 21;14(1):13. doi: 10.3390/biom14010013 (PMC10813755; doi:10.3390/biom14010013)
Supplement: Supplementary file 1 [file biomolecules-14-00013-s001.zip › biomolecules-2715541-SI.pdf]

TableS1

oxidative stress-related mitochondrial genes
CYC1
SDHB
COQ7
SDHA
UQCRC1
COQ5
PDHA1
COQ9
MRPL12
COX5A
ISCA2
PMPCB
UQCRFS1
ATP5F1A
OGDH
PDHB
UQCRC2
SDHD
MRPS35
UQCRQ
MRPL53
DBT
PDK4
MDH2
MRPS27
CS
GRPEL1
DLAT
LRPPRC
DLST
PDHX
GFM1
MPC2
NDUFS1
MRPL46
ATP5F1E
SLC25A3
MRPS23
FH
PMPCA
ATP5F1B

SDHAF4
UQCR10
ISCA1
SUCLA2
COQ3
IARS2
IDH3A
COX11
ETFDH
TIMM10
MRPL34
MRPL2
BCKDHA
UQCRH
HIGD2A
ATP5PO
ECHS1
COX6A1
COX15
AFG3L2
HADHA
ETFA
NDUFS7
CPT2
BCKDHB
IDH3B
LARS2
ACADS
LETM1
ATP5ME
OPA1
SUCLG1
NDUFV2
COQ6
MRPL43
ATP5PF
NDUFB8
LONP1
DLD
AIFM1
APOOL
MRPL10
NDUFS8
ACADM

IMMT
TIMM9
SLC25A4
SAMM50
NDUFS2
NDUFV1
ACO2
SUPV3L1
FECH
MTIF2
HIBCH
MRPS2
HSPA9
SURF1
PRDX3
GUF1
TIMM13
LYRM4
MRPL16
MRPL40
IDH3G
SDHC
NDUFB5
SDHAF2
COQ10A
TXN2
MRPS18A
COX6C
NDUFB9
MTCH2
NDUFA6
SLC25A20
MRPL1
TIMM44
COX17
MICOS10
SOD2
COX6B1
VDAC1
CLPP
HADH
ACADL
ACAT1
CLPX

NDUFS4
MRPL4
C1QBP
PITRM1
UQCC1
MRM1
MECR
MRPL44
HSDL2
MRPS14
ABCB8
ATPAF2
NDUFA9
COA6
POLRMT
ISCU
RTN4IP1
OGDHL
ATP5F1C
BCKDK
GFM2
NDUFAF4
SLC25A11
IBA57
PHB2
DAP3
SDHAF1
MRPS17
NFS1
NDUFAF5
TACO1
MFN1
PPTC7
MRPL11
COX4I1
NDUFS6
MRPL13
OXCT1
COX5B
PDK2
ALAS1
MRPL33
MTO1
LIAS

NDUFA5
NDUFB6
MTX2
SUCLG2
FDX1
SLC25A1
MRPS28
TIMM50
VDAC2
ECH1
GATB
BCS1L
ERAL1
CMC1
MRPL28
TSFM
FXN
NFU1
YARS2
MRPL21
ALDH4A1
NDUFAF1
NDUFB10
ACADSB
MRPS9
COQ8A
ACADVL
COQ4
MRPL15
NDUFA2
MRPL24
HSPD1
NDUFS5
AK3
CYCS
MIPEP
CRAT
PCCB
MRPS7
MRPL3
PRODH
PCCA
MCCC1
MRPS12

CLPB
PDK1
MRPL49
COX7A2
TMEM126A
HCCS
HIBADH
MRPL19
MRPL36
SLC25A30
BDH1
FARS2
ABCB7
MTX1
NDUFA7
ALDH9A1
MRPS18C
ALDH6A1
FDXR
GATC
TRAP1
ACAD8
ALDH2
PPIF
TIMM22
IVD
L2HGDH
ETHE1
SLC25A5
SLC25A12
MRPS21
TOMM22
ACAA2
DNAJA3
MRPS34
ETFB
ATP5IF1
ATP5PB
COX7C
CHCHD3
COQ10B
ACSF3
POLDIP2
SLC25A10

SLC25A13
PDK3
ME3
MRPL22
IDH2
GCDH
MRPL47
PPA2
MRPL9
CHCHD10
WARS2
SLC25A19
CBR4
SMDT1
HAGH
COX7A1
MTG1
COX14
NDUFA12
MRPS16
GPD2
NDUFA10
NDUFC2
SLIRP
MT-ATP6
MT-CO2
NDUFA8
GLS
NDUFB11
DHTKD1
HINT2
MRPS5
DECR1
MRPS26
SLC25A15
NDUFV3
BPHL
STOML2
MSRB2
LACTB
TOMM40
SLC25A25
ACAD10
DNAJC11

ALKBH7
GLDC
THEM4
SSBP1
HSCB
MRPS10
AK4
ATAD3A
MRPL37
PTGES2
TXNRD2
CISD1
MRPL41
MTARC2
MRPS6
MRPL23
SQOR
SCO1
GCAT
MTHFD1L
ECI2
UQCRB
ATP5PD
ATP5MG
MCCC2
MCEE
MOCS1
MMUT
SPG7
ATP5MC3
ATAD1
DARS2
MRPL51
PC
PNPLA8
MLYCD
MRPL32
PRODH2
ME2
TUFM
COX6A2
SLC30A9
TAMM41
TIMM23

MFN2
CHCHD4
NDUFAB1
ACSM1
COQ2
PDSS2
EHHADH
NDUFS3
ABCB10
CKMT2
MRPS25
ACAD9
COX16
FAM210A
ACOT13
CPT1A
DHRS4
CARS2
NIPSNAP2
NDUFB7
MICOS13
GLRX2
MRPS30
SCO2
HSD17B10
NARS2
RPUSD3
SUOX
SARDH
COX8A
SPR
MPV17
MRPL18
ALDH1B1
CHCHD1
TIMM8A
VDAC3
SLC25A24
MPC1
SLC25A22
SLC25A31
TRNT1
IDE
GSTK1

VARS2
HSPE1
MRPL38
MTCH1
TRIAP1
PTCD3
COX10
AKAP1
AK2
TMEM70
NOA1
MCUR1
NDUFC1
MRPL35
NDUFA3
PDSS1
ACSM3
NDUFAF6
UCP1
SFXN1
GLRX5
MRPS11
XPNPEP3
PDP1
ECI1
SLC25A21
SLC25A29
SDHAF3
SLC25A39
REXO2
GATM
SARS2
C8orf82
EARS2
GRHPR
MTFMT
ECHDC1
HARS2
NDUFA4
NDUFA13
TMEM11
SELENOO
NAGS
HSD17B4

NDUFAF7
OAT
MRPL50
COX7B
RARS2
NDUFA11
CHCHD7
SHMT2
MTHFD2
RMDN1
CPS1
MRPL14
MTRES1
RDH13
MRPS24
GSR
ENDOG
BCAT2
TEFM
ACSF2
TRMU
MRPS22
LAP3
GLUD1
HMGCL
FHIT
MPV17L
GADD45GIP1
TFAM
ALDH18A1
OXSM
KYAT3
TARS2
TBRG4
ROMO1
PRDX5
DNAJC15
HIGD1A
RHOT2
SLC25A44
OXA1L
SLC25A46
RDH14
CA5B

COASY
CISD3
GSTZ1
ACAA1
COX19
PDPR
NLN
BOLA1
ALAS2
GRPEL2
ALDH7A1
GFER
IMMP2L
COX4I2
MRPL39
FUNDC2
LYPLA1
PDHA2
GTPBP3
YME1L1
ACOT2
COA8
MMAB
PPOX
ABCD3
SIRT3
LDHD
MRPL45
POLG
ATPAF1
BLOC1S1
ALDH1L2
CPOX
NNT
ATP5MF
SFXN3
AASS
DIABLO
AGXT2
GLS2
APOO
ACAD11
TTC19
NGRN

GCSH
SIRT5
ABCB6
MACROD1
TST
MT-CO1
SDR39U1
DMGDH
SLC25A14
CHCHD5
TOMM70
MCU
CYP27A1
MT-ND2
MT-ND4
MT-ND5
MRPS31
ALDH1L1
PTCD1
HADHB
TRIT1
CRLS1
SLC25A32
AARS2
EXOG
CHCHD2
KMO
SFXN5
ELAC2
GPX1
CPT1B
FIS1
PRELID1
UCP3
SLC25A27
SLC25A40
SLC25A23
OMA1
DHRS1
STOM
MSRA
NMNAT3
SLC25A38
MPV17L2

ACLY
ABCD2
FLAD1
LIPT1
PCK2
NADK2
MPST
ACACA
ABAT
HMGCS2
ALDH5A1
CKMT1A
PISD
NDUFB4
TOMM5
HTRA2
NDUFA1
MT-CYB
GUK1
D2HGDH
OPA3
AKR1B10
PHYH
NDUFAF2
ECSIT
PARL
MRPL42
DNAJC4
PAM16
ALDH3A2
ABHD10
OSGEPL1
NUDT2
CYB5B
GRSF1
MRPS18B
DNAJC30
CAT
DGUOK
ACOT9
ACSS1
RFK
STARD7
CHPT1

MT-ATP8
TIMMDC1
HTATIP2
AKR7A2
DUT
MT-CO3
DHRS7B
MTRF1
RHOT1
NME4
TIMM29
PNPO
MICU2
PCBD2
QRSL1
PRDX6
NIPSNAP1
GARS1
PARS2
MRPL52
PUS1
GOT2
MT-ND3
ACCS
PTRH2
PGS1
OXR1
FPGS
AIFM3
MGST1
NIF3L1
BAX
MAVS
METTL17
ACACB
ARG2
MTPAP
CCDC51
SUGCT
DNM1L
TMEM126B
NSUN4
ACSS3
AHCYL1

MTARC1
FUNDC1
ACSL1
COX20
HSDL1
CBR3
MTERF4
NUDT19
PTPMT1
PGAM5
DTYMK
KARS1
STAR
DNAJC19
OTC
COX7A2L
MTERF3
FASN
PINK1
TMEM177
TXNRD1
THNSL1
FDPS
IDI1
FTMT
LDHAL6B
MTFR1
BCL2L13
TRMT10C
ACOT7
TFB1M
TWNK
MMADHC
PYCR2
RECQL4
PET100
GDAP1
MTIF3
UNG
GLYAT
ATP5MC1
QDPR
COQ8B
GTPBP10

TSPO
FTH1
TRMT1
MRPS36
AGPAT5
MCRIP2
UCP2
SLC25A28
EPHX2
NUDT5
MICU1
SOD1
NDUFAF3
BOLA3
CHCHD6
FASTKD2
PEX11B
PIF1
FABP1
ABCD1
DBI
PNPT1
TOP1MT
RNASEH1
SCP2
LDHB
HINT1
MUL1
TK2
AGMAT
BID
SYNJ2BP
MFF
DCXR
NUDT9
AMACR
NUDT6
MRM2
BAK1
DHODH
MAOB
MGST3
MRPL48
AGXT

CYB5R3
DDX28
PNKD
BNIP3
MUTYH
GTPBP6
CYP11A1
OCIAD1
NME6
FKBP8
NLRX1
FOXRED1
TFB2M
PPM1K
MT-ND1
MSRB3
ACOT11
NTHL1
MARCHF5
SFXN4
TRMT5
SLC25A6
OGG1
ACOD1
AIFM2
PRDX2
FBXL4
GPAM
RMDN3
PRXL2A
MAOA
POLQ
BCO2
IFI27
CRYZ
PYCR1
TMLHE
TOMM20
DHRS2
MMAA
MIEF1
C15orf48
LIG3
BNIP3L

FASTKD5
PARK7
COA1
MT-ND4L
SIRT4
UQCC2
USP30
PDE12
NME3
DHX30
CYP24A1
PANK2
CA5A
NT5DC2
ATAD3B
TRUB2
PRKACA
COMTD1
CPT1C
SND1
GLUD2
HPDL
UQCC3
PRDX4
CDK5RAP1
PAICS
CYP11B2
AGPAT4
RCC1L
PMAIP1
TDRKH
CYP27B1
POLG2
BIK
MTERF1
GPX4
COMT
SPATA20
CRY1
MGME1
SEPTIN4
EFHD1
RAB5IF
APEX1

SPTLC2
CASP9
AGK
MCL1
DUS2
PLD6
TOMM34
PRORP
CKMT1B
CASP8
BCL2L1
EXD2
ARMC10
MIEF2
THG1L
GOLPH3
PREPL
CEP89
NBR1
BOK
FKBP10
CYP11B1
CSKMT
RPIA
BBC3
BAD
BCL2
BCL2A1
BCL2L2
CASP3
DMPK
DNA2
MT-ND6
PRKN
ALKBH1
PICK1
BCL2L10
STYXL1
NSUN2
ADCY10
SPHK2
PRIMPOL
STX17
PDE2A

BCL2L11
SPIRE1
C2orf69
POLB
SNAP29
ARMCX6
SPHKAP

R code:

We will provide a portion of the R code in the supplementary materials. If a complete version is needed, please inform us, and we will make the necessary arrangements.

```
library(limma)
library(sva)
mergeFile="merge.preNorm.txt"
normalizeFile="merge.normalzie.txt"
setwd()
files=c()
geneList=list()
for(i in 1:length(files)){
	fileName=files[i]
	rt=read.table(fileName, header=T, sep="\t", check.names=F)
	header=unlist(strsplit(fileName, "\\.|\\-"))
	geneList[[header[1]]]=as.vector(rt[,1])
}
intersectGenes=Reduce(intersect, geneList)
allTab=data.frame()
batchType=c()
for(i in 1:length(files)){
	fileName=files[i]
	header=unlist(strsplit(fileName, "\\.|\\-"))
	rt=read.table(fileName, header=T, sep="\t", check.names=F)
	rt=as.matrix(rt)
	rownames(rt)=rt[,1]
	exp=rt[,2:ncol(rt)]
	dimnames=list(rownames(exp),colnames(exp))
	data=matrix(as.numeric(as.matrix(exp)), nrow=nrow(exp), dimnames=dimnames)
	rt=avereps(data)
	colnames(rt)=paste0(header[1], "_", colnames(rt))
	qx=as.numeric(quantile(rt, c(0, 0.25, 0.5, 0.75, 0.99, 1.0), na.rm=T))
	LogC=( (qx[5]>100) || ( (qx[6]-qx[1])>50 && qx[2]>0) )
	if(LogC){
	  rt[rt<0]=0
```

```
        rt=log2(rt+1)}
    rt=normalizeBetweenArrays(rt)
    if(i==1){
      allTab=rt[intersectGenes,]
    }else{
      allTab=cbind(allTab, rt[intersectGenes,])
    }
    batchType=c(batchType, rep(header[1],ncol(rt)))
}
allTabOut=rbind(geneNames=colnames(allTab), allTab)
write.table(allTabOut, file=mergeFile, sep="\t", quote=F, col.names=F)
normalizeTab=ComBat(allTab, batchType, par.prior=TRUE)
normalizeTab=rbind(geneNames=colnames(normalizeTab), normalizeTab)
write.table(normalizeTab, file=normalizeFile, sep="\t", quote=F, col.names=F)
```
